# Stromal Claudin14-Heterozygosity, but Not Deletion, Increases Tumour Blood Leakage without Affecting Tumour Growth

**DOI:** 10.1371/journal.pone.0062516

**Published:** 2013-05-13

**Authors:** Marianne Baker, Louise E. Reynolds, Stephen D. Robinson, Delphine M. Lees, Maddy Parsons, George Elia, Kairbaan Hodivala-Dilke

**Affiliations:** 1 Centre for Tumour Biology, Barts Cancer Institute - a CR-UK Centre of Excellence, Queen Mary University of London, London, United Kingdom; 2 School of Biological Sciences, University of East Anglia, Norwich, United Kingdom; 3 Randall Division of Cell and Molecular Biophysics, Kings College London, London, United Kingdom; Lerner Research Institute, United States of America

## Abstract

The maintenance of endothelial cell-cell junctions is vital for the control of blood vessel leakage and is known to be important in the growth and maturation of new blood vessels during angiogenesis. Here we have investigated the role of a tight junction molecule, Claudin14, in tumour blood vessel leakage, angiogenesis and tumour growth. Using syngeneic tumour models our results showed that genetic ablation of Claudin14 was not sufficient to affect tumour blood vessel morphology or function. However, and surprisingly, Claudin14-heterozygous mice displayed several blood vessel-related phenotypes including: disruption of ZO-1-positive cell-cell junctions in tumour blood vessels; abnormal distribution of basement membrane laminin around tumour blood vessels; increased intratumoural leakage and decreased intratumoural hypoxia. Additionally, although total numbers of tumour blood vessels were increased in Claudin14-heterozygous mice, and in VEGF-stimulated angiogenesis *ex vivo*, the number of lumenated vessels was not changed between genotypes and this correlated with no difference in syngeneic tumour growth between wild-type, Claudin14-heterozygous and Claudin14-null mice. Lastly, Claudin14-heterozygosity, but not complete deficiency, also enhanced endothelial cell proliferation significantly. These data establish a new role for Claudin14 in the regulation of tumour blood vessel integrity and angiogenesis that is evident only after the partial loss of this molecule in Claudin14-heterozyous mice but not in Claudin14-null mice.

## Introduction

Blood vessel maturation, during angiogenesis, involves establishment of endothelial cell-cell contacts, deposition of an intact basement membrane, lumen formation and the recruitment of supporting cells [1]–[3]. The quality of tumour blood vessels differs significantly from the normal vasculature. Their rapid formation leads to increased vessel leakage, poorly controlled lumen formation and discontinuous supporting cell association. Tumour blood vessels are characteristically leaky, thus enhancing the diffusion of oxygen and nutrients into the tumour mass, but their chaotic organisation also results in regions of intratumoural hypoxia and necrosis [4], [5].

Endothelial cell-cell junction integrity is a determinant of vascular permeability. Tight junctions (TJs) in both epithelial and endothelial cells consist of several protein families, including JAMs, occludin and claudins [6]. TJs are signal transduction complexes that communicate information from the environment to the cell interior, regulating cell morphology, motility, gene expression, signalling and crosstalk with non-TJ proteins [7]. The regulation of TJ formation [8], [9] can impact upon several cellular processes, including vascular permeability and cell proliferation [10], [11].

Some TJ protein families have endothelial cell-specific members such as vascular endothelial junctional adhesion molecule (VE-JAM, also known as JAM2 or JAM-B), and endothelial cell-enriched molecules, for example Claudin5 (Cldn5) [12], [13]. Cldn5 is typically thought to be the major endothelial claudin protein, but its expression has now also been noted in rat pancreatic acinar cells, stomach and gut epithelia, and in human immune cells [13]–[15]. It is known to be vital in maintaining the integrity of the endothelial blood-brain barrier (BBB), since knockout mice display size-selective loosening of the barrier [16]. Cldn5 expression appears to be dependent on the adhesion of endothelial cells to the ECM via β1-integrin, and is controlled by adherens junctions via VECAD, FoxO1 and β-catenin transcriptional regulation [17]–[19].

Another claudin family member, Claudin14 (Cldn14), has been identified in both epithelial and endothelial cell layers. Cldn14 mutations have been identified as a key cause of autosomal recessive deafness [20], [21]. Additionally this molecule has been found to be a regulator of kidney epithelial permeability [22]. However, the precise functions of Cldn14 in angiogenesis *in vivo* and particularly in tumour angiogenesis are unknown.

Here we describe that, surprisingly, homozygous loss of Cldn14 has little to no effect on tumour angiogenesis. However, in Cldn14-heterozygous mice (with loss of only one copy of Cldn14) we found the following major phenotypes: (1) disruption of cell-cell junctions in tumour blood vessel; (2) abnormal tumour blood vessel basement membrane organisation and reduced supporting cell association; (3) increased intratumoural leakage and decreased tumour hypoxia; (4) enhanced tumour angiogenesis but no significant difference in the proportion of lumenated tumour blood vessels; (5) no effect on syngeneic tumour growth and (5) increased endothelial cell proliferation *in vivo*, *ex vivo* and *in vitro*.

In short our data establish that Cldn14 heterozygosity, but not complete deficiency, can affect tumour blood vessel functionality and describe a gene dosage effect of this molecule on angiogenic processes.

## Methods

### Mice

All animals were used in accordance with UK Home Office regulations and approved by the Queen Mary University of London and Oxford University ethics committee. License 70/7449.

Claudin14-null mice on a C57/bl6 genetic background [20] were crossed with pure C57/bl6 (wild-type or WT) mice purchased from Charles River to create heterozygous (Het) progeny, from which new breeding pairs were set up to create colonies of Cldn14 WT, Cldn14-heterozygous and Cldn14-null mice. Genotyping was performed using the common primer: Cldn14 common [5′ - GGC TGC ATA ACC AGG ATA CTC - 3′] with Cldn14 WT primer [5′ - GTA CAG GCT GAA TGA CTA CGT G - 3′] for the wild-type allele (340 bp band) and Cldn14 common with Cldn14 Mutant primer [5′ - CAG CTC ATT CCT CCC ACT CAT GAT C - 3′] for the null allele (275 bp band) in two separate PCR reactions. Mice were born in normal Mendelian ratios and male:female ratios, no obvious adverse effects were observed, and proportionately decreased Cldn14 mRNA levels were confirmed in kidneys and brains of Cldn14-het and Cldn14-null animals, compared to wild-type littermates (see **Figure S1**).

### Subcutaneous syngeneic tumour models

Mouse tumour cell lines B16F10 melanoma (ATCC® Number: CRL-6475 ™) and Lewis Lung Carcinoma (ATCC® Number: CRL-1642 ™) cells were grown in DMEM (Gibco) supplemented with 10% v/v FBS (EU-approved heat-inactivated fetal bovine serum (PAA Laboratories, A15–104) and penicillin/streptomycin (Gibco). Tumour cells were trypsinised and resuspended in PBS at a concentration of 5×10^6^ cells ml^−1^. 0.5×10^6^ tumour cells in 100 µl PBS were injected subcutaneously in the flank. Palpable tumours were measured with callipers every other day and bisected. Half of the tumour was snap-frozen for cryosectioning, and the other half fixed in 4% formalin. Formalin-fixed tumours were embedded in paraffin for sectioning.

### Antibodies

Anti-endomucin 1∶500 (Santa Cruz: V.7C7 sc-65495); anti-α-Smooth Muscle Actin-Cy3 1∶1000 (Sigma, C6198); anti-Pimonidazole 1∶10 (HPI, Inc. HP2-1000); Ki67 1∶100 (Vector Labs, VPK451), anti-ZO-1 1∶100 (Invitrogen, 40–2200). PE-PECAM 1∶500 (Biolegend, 102408), FITC-BS1 lectin (Sigma Isolectin B_4_, L2895).

### Hoechst leakage assay

10 minutes prior to sacrifice, tumour-bearing mice were injected with 100 µl PE-PECAM antibody (undiluted, BioLegend: 102408). 1 minute before sacrifice, the same mice were injected intravenously in the tail vein with 100 µl 4 µg/ml Hoechst dye (Sigma bisBenzimide H33342 trihydrochloride, B2261), diluted in ddH_2_O. Tumours were processed immediately after cervical dislocation. 100 µm cryosections were thawed, rehydrated and fixed for 10 minutes in −20°C methanol then mounted with ProLong Gold™ with Antifade (Invitrogen, P36930). 100 µm Z-stacks (stack interval 0.5 µm, 20× magnification) were taken using a Zeiss LSM 510 confocal microscope. LSM images were analysed using ImageJ: red (PE-PECAM) and blue (Hoechst) pixel intensities were obtained using colour thresholding to remove background noise and Hoechst staining was quantified and normalised to blood vessel density for each section to give Relative Intensity values.

### Pimonidazole detection of hypoxia

1 hour prior to sacrifice, tumour-bearing mice were injected with 60 mg/kg pimonidazole hydrochloride (Hypoxyprobe™-1 HPI, Inc., diluted in ddH_2_O to a final concentration of 10 mg/ml) intravenously via the tail vein [23]. Tumours were processed immediately after cervical dislocation. 8 µm cryosections were thawed, rehydrated and fixed for 10 minutes in −20°C acetone then incubated with 1∶10 anti-pimonidazole and 1∶500 PE-conjugated anti-PECAM antibodies to identify hypoxic areas and blood vessels respectively. Sections were then washed and mounted with ProLong Gold™ with Antifade (Invitrogen, P36930). Images were taken with a Zeiss microscope and Axioplan camera. Grids were used to divide the images into sectors and the distance from PECAM-positive blood vessels to the closest pimonidazole-positive (hypoxic) areas were measured in Adobe Photoshop CS5. The average inverse values of these distances were taken to give the hypoxic index.

### Immunohistochemistry

FFPE 8 µm sections were processed as follows: (1) antigen retrieval 2×10 minutes boiling in citrate buffer (0.294% w/v Tri-Sodium citrate (Fisher) in ddH_2_O, brought to pH 6 with acetic acid). (2) Deparaffinising in xylene and ethanol solutions of decreasing concentration. (3) Blocking: 30 minutes in 2% v/v Goat serum, 1% w/v Bovine Serum Albumin, 0.1% v/v Triton X-100 in PBS. (4) Washing with 0.1% w/v Bovine Serum Albumin in PBS. (5) Incubation with anti-endomucin primary antibody (diluted in wash buffer) overnight at 4°C. (6) Washing in wash buffer. (7) Incubation with 1∶100 secondary antibodies (Invitrogen: Alexa Fluor® 488, with addition of α-SMA-Cy3 for pericytes). (8) Final washing with PBS then ddH_2_O. (9) Mounting with ProLong® Gold Antifade with DAPI (Invitrogen: P36931).

8 µm cryosections were processed for ZO-1 and PECAM immunostaining as follows: 1) fixation with −20°C methanol for 10 minutes. 2) Blocking: 45 minutes in 5% w/v BSA, 0.1% v/v Triton X-100 in PBS. 3) 3× PBS washes. 4) Incubation with primary antibodies for 45 minutes at room temperature, diluted in blocking buffer. 5) 3× PBS washes. 6) Incubation with secondary antibodies for 2 hours, diluted in blocking buffer. 7) 3× PBS washes. 8) Final H_2_O wash. 9) Mounting with ProLong® Gold Antifade with DAPI.

8 µm cryosections were processed for Ki67 immunostaining as follows: 1) fixation in −20°C acetone for 10 minutes. 2) Permeabilisation with 0.5% NP-40 for 10 minutes. 3) Blocking: 1 hour in 1% w/v BSA, 0.1% Triton X-100 in PBS. 4) Incubation with primary antibody diluted in blocking buffer at 4°C overnight. 5) Washing, secondary antibody incubation and mounting as in steps 5–9 above.

### Aortic Ring Assay

The aortic ring assay was performed as described in Baker *et al*. 2012 [24]. Briefly, thoracic aortae were taken from mice sacrificed by cervical dislocation then cleaned and cut into 1 mm thick rings. Rings were serum-starved overnight, embedded in a collagen-OptiMEM® mixture and fed with OptiMEM® containing 2.5% FBS (EU-approved heat-inactivated fetal bovine serum (PAA Laboratories, A15–104) and 30 ng/ml VEGF or PBS as a control. Endothelial microvessel sprouts were counted every other day from days 5–9 until fixation of the explant cultures using 4% formalin. Following fixation, the cultures were stained with FITC-conjugated BS-1 Lectin (Sigma, L9381) and Cy3-conjugated α-SMA antibody then mounted on slides using ProLong Gold™ with Anti-fade and DAPI. A Zeiss AxioPlan microscope and AxioVision software were used for imaging. For the Invitrogen ClickIT® EdU proliferation assay [24]; aortic rings are treated with EdU prior to fixation as previously described and then stained with Alexa Fluor® 488 secondary antibody as according to the manufacturers' protocol, using reduced reagent volumes. Rings are co-stained with TRITC-conjugated BS1-lectin (Sigma, L5264).

### Primary endothelial cell culture

Primary mouse endothelial cells were purified and cultured from mouse lung as described in Reynolds and Hodivala-Dilke, 2006 [25]. Briefly, mouse lungs were dissected, rinsed in F-12 + GlutaMAX™ (Gibco) medium, 70% ethanol then MLEC medium (1∶1 mixture of F-12 and low-glucose DMEM (DMEM + GlutaMAX™ +1 g/L D-Glucose + Pyruvate, Gibco) supplemented with 20% v/v FBS, 100 mg L^−1^ heparin (Sigma), 1% v/v 100× glutamine (GlutaMAX™ 100×, Gibco 35050), Endothelial Growth Supplement (AbD Serotec, 4110–5004) and 1% v/v 100× penicillin/streptomycin (Gibco)). Lungs were then minced and digested in 0.1% Type I Collagenase (Gibco, 17100–017), passed through a 70 µm cell strainer (BD Falcon) and the resulting single-cell suspension plated on pre-coated plastic. Macrophages were removed from the resulting cultures using rat anti-mouse FCγII/III (Pharmingen) antibody and sheep anti-rat IgG-coated magnetic bead sorting (Dynal). Endothelial cell cultures were enriched by positive sort, using two rounds of anti-CD102 (ICAM2) antibody (Pharmingen) and Dynal anti-rat magnetic beads.

## Results

### Cldn14 heterozygosity, but not deficiency, destabilises tumour blood vessel morphology

It is conceivable that Cldn14 could affect vascular integrity, since it is a component of tight junctions. To test the requirement for Cldn14 in the maintenance of vascular integrity and in angiogenic processes *in vivo*, we used a Cldn14 genetic ablation approach. Cldn14-heterozygous (Cldn14-het) mice were inter-crossed to produce wild-type (WT), Cldn14-het and Cldn-14 null progeny. No effects on Mendelian ratios or male:female ratios were observed in litters (**Figure S1** **A**–**C**). qPCR analysis confirmed that levels in Cldn14-het organs were approximately half those detected in WT mice, whilst Cldn14 transcript was undetectable in Cldn14-nulls (**Figure S1 D**). Due to the lack of reliable reagents, we were not able to test whether Cldn14 is expressed differentially in quiescent and angiogenic blood vessels *in vivo*.

**Figure 1:**
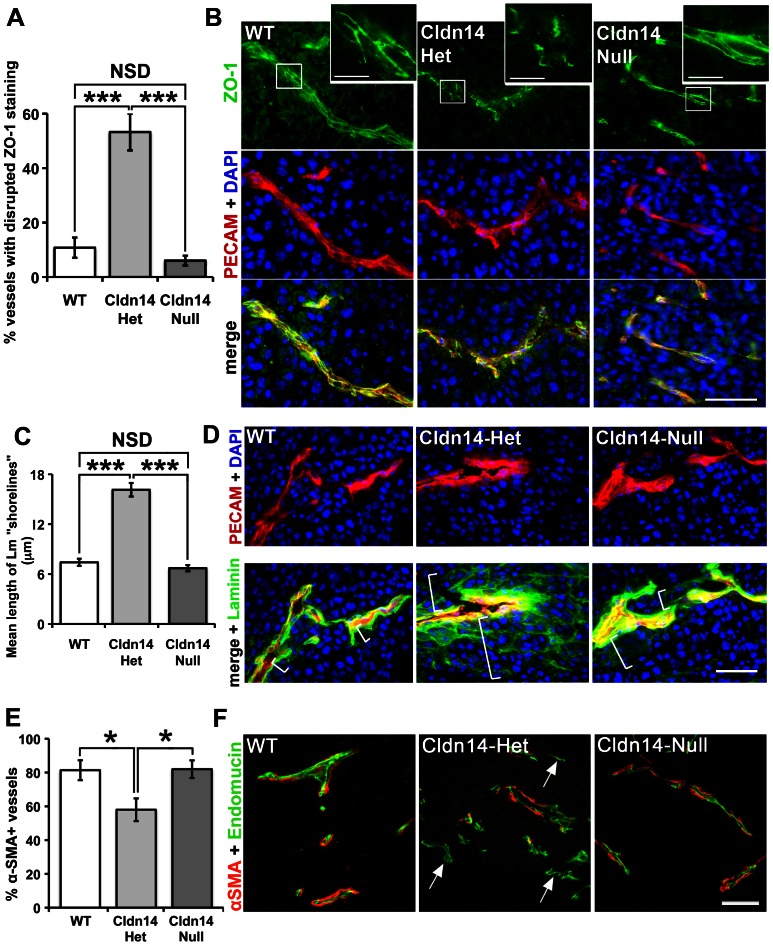
Cldn14 heterozygosity destabilises tumour blood vessels. B16F10 tumours were grown for 10 days in WT, Cldn14-het and Cldn14-null mice and midline sections from size-matched tumours were analysed for blood vessel stabilisation by immunostaining for the tight junction component ZO-1, basement membrane laminin, and pericyte coverage using an anti-αSMA antibody. (**A**) The tight junction adapter protein ZO-1 staining pattern was observed at cell-cell borders in PECAM-positive tumour blood vessels. A higher proportion of blood vessels exhibited a disrupted ZO-1 staining pattern in Cldn14-het tumour sections, compared to tumours from WT and Cldn14-null mice. (**B**) Representative images of ZO-1 staining and the endothelial cell marker PECAM, with nuclei DAPI-counterstained. Inserts show higher magnification of ZO-1 at cell-cell junctions. Scale bars: main panels  = 50 µm, insets  = 10 µm. (**C**) The AxioVision software linear measuring tool was used to analyse the spread (in µm) of laminin surrounding PECAM-positive blood vessels in immunostained tumour sections. Laminin expression was close to blood vessel walls in tumours from WT and Cldn14-null mice but disorganised around tumour blood vessels in Cldn14-het mice; a “shorelining” effect of laminin deposition was evident in these sections. (**D**) Representative images of tumour cryosections immunostained for basement membrane laminin and the endothelial cell marker PECAM, with nuclei DAPI-counterstained. *White brackets* indicate the spread of laminin staining radiating from PECAM-positive vessels and demonstrates the quantification method. (**E**) α-Smooth Muscle Actin (αSMA) antibody conjugated to Cy3 fluorescent dye was used to label supporting cells (pericytes) around endomucin-labelled blood vessels in midline tumour sections. The percentage of αSMA-positive vessels was quantified. (**F**) Representative images of endomucin and αSMA double-stained tumour sections. *Arrows*, αSMA-negative vessels. Scale bar 50 µm. N = 4–6 tumours per genotype. Bar charts represent means ± SEM. * *P*<0.05, *** *P*<0.001.

Given that Cldn14 is a tight junction protein, we first asked whether deletion of Cldn14 could affect tumour endothelial cell-cell junction organisation. Wild-type (WT), Cldn14 heterozygous (Cldn14-het) and Cldn14-null mice were injected subcutaneously with 0.5×10^6^ B16F10 melanoma cells. At 10 days post tumour cell inoculation the tumours were snap-frozen and cryosections double immunostained for the tight junction marker ZO-1 and the endothelial cell marker PECAM. ZO-1 staining was confined to a continuous pattern of expression at endothelial cell-cell junctions in tumours from both WT and Cldn14-null animals, indicating that Cldn14-deletion was not sufficient to affect the organisation of ZO-1 at cell-cell junctions. In contrast, and surprisingly, a discontinuous staining pattern of ZO-1 was observed in the majority of Cldn14-het tumour blood vessels (**Figure 1A, B**). This suggests that ZO-1 localisation to endothelial tight junctions is affected by partial but not total loss of Cldn14 in blood vessels and that this may contribute to the functionality of these vessels.

Given that extracellular matrix deposition and maintenance are crucial steps in the maturation of tumour blood vessels, we next asked if the distribution of laminin within the blood vessel basement membrane was affected by changes in stromal Cldn14 levels. Tumour sections were double immunostained for PECAM and laminin and observations showed that the pattern of laminin deposition was proximate to the blood vessel wall in sections from WT and Cldn14-null mice, indicating, again that Cldn14 deficiency was not sufficient to affect this process. In contrast, a high frequency of disorganised laminin deposition around blood vessels, with a ‘shorelining’ pattern, was observed in sections from tumours grown in Cldn14-het mice (**Figure 1C, D**). This result showed that the organisation of the basement membrane might be affected by partial loss of Cldn14.

Blood vessel stabilisation is a consequence not only of basement membrane deposition, but also the association of supporting α-SMA-positive cells [26], [27]. To assess this, tumour sections were co-stained for the differentiated pericyte cell marker α-SMA and for endomucin. Results showed that the proportion of blood vessels with α-SMA-positive pericyte association was reduced significantly in tumours grown in Cldn14-het mice (**Figure 1E, F**).

### Stromal Claudin14-heterozygosity enhances tumour blood vessel Hoechst leakage and decreases tumour hypoxia

**Figure 2:**
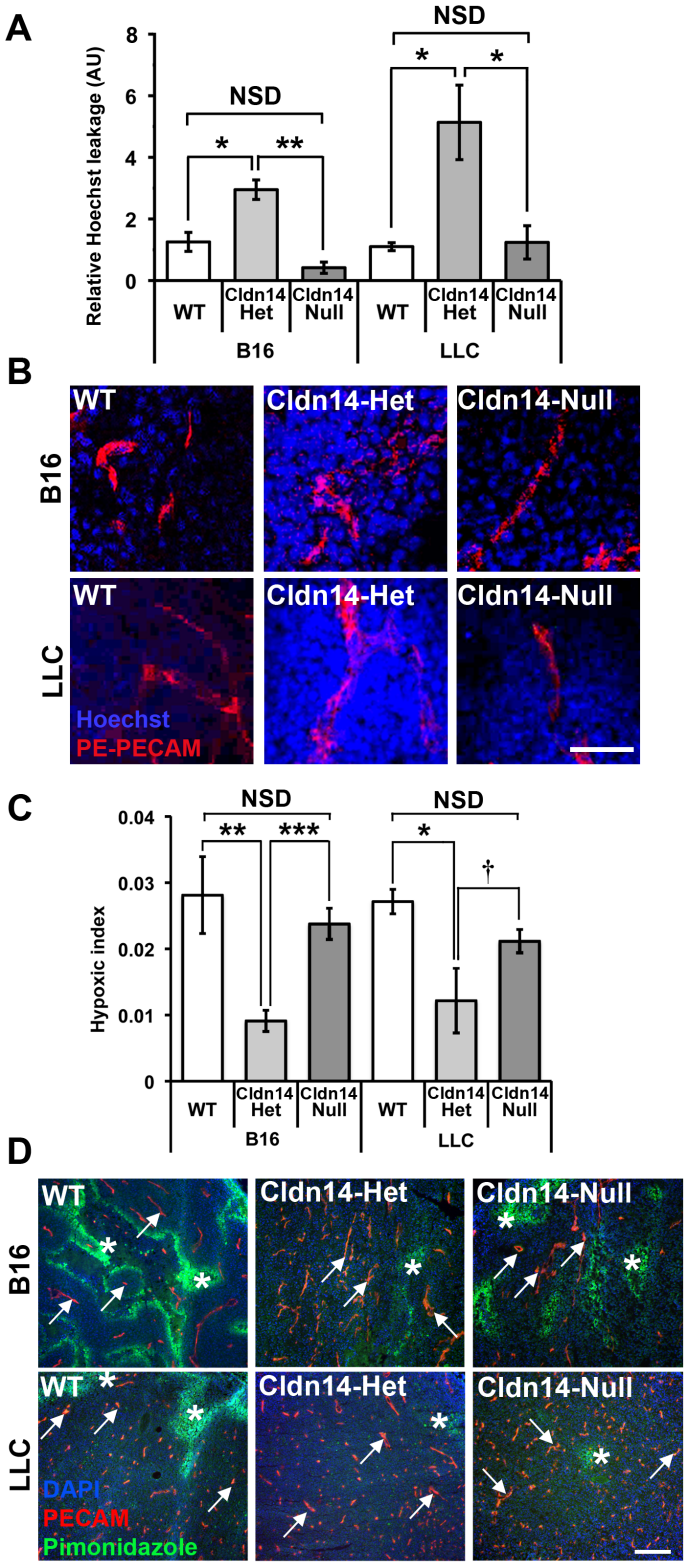
Heterozygosity for Cldn14 increases tumour blood vessel leakage and decreases intratumoural hypoxia. Wild-type, Cldn14-heterozygous and Cldn14-null mice were injected subcutaneously in the flank with 0.5×10^6^ B16F10 melanoma or Lewis Lung Carcinoma (LLC) cells. (**A**) At 10 days post inoculation, PE-conjugated anti-PECAM antibody and Hoechst dye were injected via the tail vein prior to sacrifice. Midline sections (100 µm) of snap-frozen tumours were fixed, mounted and imaged using a Zeiss LSM 510 confocal microscope. The extent of Hoechst leakage was measured in z-stacks using ImageJ. Bars show mean Hoechst leakage relative to PECAM signal ± SEM. Blood vessel leakage is increased significantly in Cldn14-het mice when compared with WT and Cldn14-null mice. (**B**) Representative images of Hoechst (blue) and PECAM (red) detection. (**C**) Tumour-bearing mice from each genotype were injected with pimonidazole prior to sacrifice to measure hypoxic areas within the tumour. 8 µm tumour cryosections were then double stained with anti-pimonidazole antibody (green) to highlight hypoxic areas and anti-PECAM antibody to identify blood vessels. The hypoxic index was quantified relative to PECAM staining using image J software. Bars represent mean relative hypoxic index ± SEM. (**D**) Representative images of pimonidazole detection and PECAM-positive blood vessels in tumour sections. *Arrows*, blood vessels; *Asterisks*, pimonidazole-positive staining. Scale bars: **A** 50 µm; **D** 200 µm. N = 4 tumours per genotype. NSD: not statistically different, * *P*<0.05, ** *P*<0.01, *** *P*<0.001, † *P* = 0.09.

Considering the possible destabilisation of tumour blood vessels in Cldn14-het animals, we sought to investigate whether loss of Cldn14 affected the leakage of tumour blood vessels. WT, Cldn14-het and Cldn14-null mice were injected subcutaneously with 0.5×10^6^ B16F10 melanoma or Lewis Lung Carcinoma (LLC) cells. Intravenous injection of anti-PECAM (PE-PECAM) antibody and Hoechst dye into tumour-burdened mice revealed no significant difference in Hoechst leakage from tumour vessels between WT and Cldn14-null tumour-bearing mice relative to PE-PECAM signal. However, a significant increase in Hoechst leakage was observed in Cldn14-het mice when compared with either WT or Cldn14-null mice (**Figure 2A and B**).

These results were corroborated when we examined the relative levels of tumour hypoxia. Tail vein injections of pimonidazole Hypoxyprobe™ (HPI, Inc.) into tumour-bearing WT, Cldn14-het and Cldn14-null mice showed that the relative levels of tumour hypoxia were similar between WT and Cldn14-null mice (**Figure 2C, D**). In contrast, hypoxic levels were found to be significantly lower in both B16F10 and LLC tumours grown in Cldn14-het mice when compared with controls (**Figure 2C, D**).

These data suggest that loss of one Cldn14 allele within the stromal compartment is sufficient to enhance tumour blood vessel leakage and decrease tumour hypoxia, but that complete Cldn14 deficiency does not affect these processes.

### Stromal Cldn14-heterozygosity does not affect tumour growth rates

**Figure 3:**
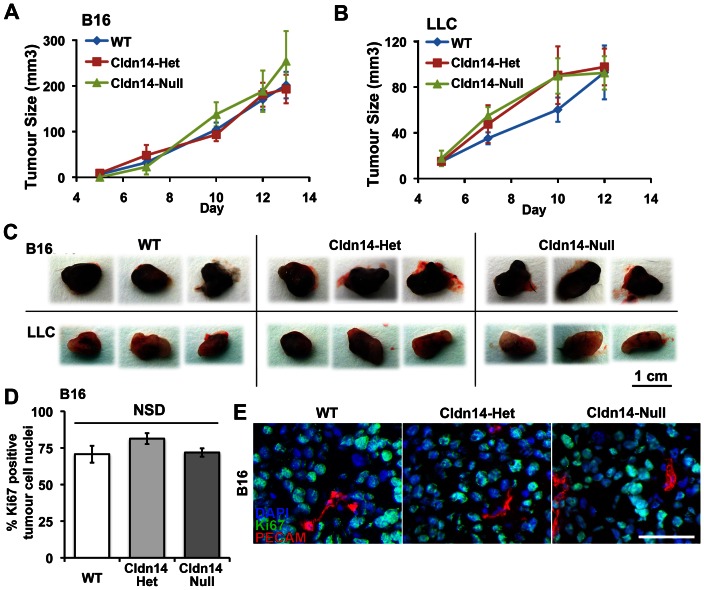
Stromal Cldn14 heterozygosity does not affect tumour size or tumour cell proliferation. Wild-type and Cldn14-het and Cldn14-null mice were injected subcutaneously with 0.5×10^6^ B16F10 melanoma or Lewis Lung Carcinoma (LLC) cells. (**A** and **B**) Tumour size was measured every two days for up to 13 days. No difference in B16 or LLC tumour growth rate was observed between the genotypes. (**C**) Representative images of B16 and LLC endpoint tumours from each genotype. N = 12–17 mice per tumour type per genotype. (**D**) Tumour cryosections were immunostained for the proliferation marker Ki67 and the endothelial marker PECAM, with a DAPI nuclear stain. The percentage of Ki67-positive/PECAM-negative tumour cells was counted. Bars show mean ± SEM. (**E**) Representative images of Ki67-stained tumour sections. Scale bar 50 µm. NSD: no significant difference.

The changes observed in tumour blood vessel leakage and hypoxia may have been indicative of changes in tumour growth in Cldn14-het mice. However, analysis of luciferase-tagged B16 and LLC tumour growth rates demonstrated no significant differences between any of the genotypes (**Figure 3A–C**). The lack of change in tumour growth corresponded with no significant differences in tumour cell proliferation in any of the genotypes (**Figure 3D, E**). Thus, despite the apparent defects in the tumour blood vessel morphology in Cldn14-het mice, no effect on tumour growth was apparent.

### Cldn14-heterozygous mice have increased tumour blood vessel density, but show no difference in the number of lumenated tumour blood vessels

**Figure 4:**
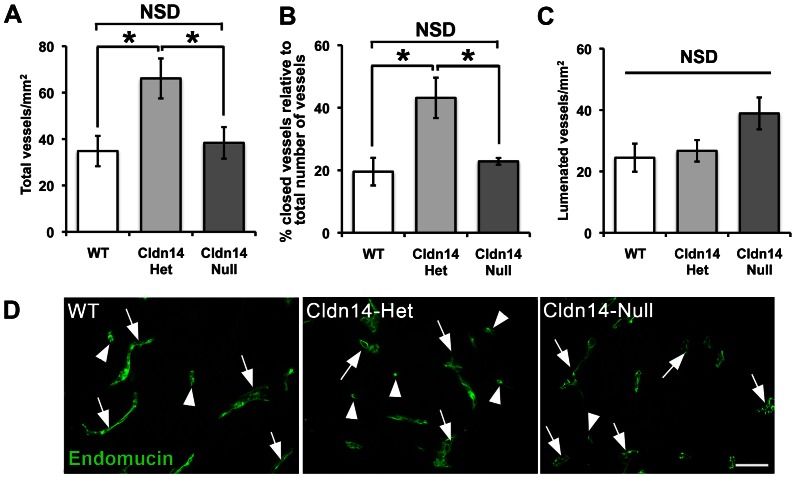
Cldn14-heterozygous mice have increased tumour blood vessel density, but show no difference in the number of lumenated tumour blood vessels. Wild-type and Cldn14-het and Cldn14-null mice were injected subcutaneously with 0.5×10^6^ B16F10 melanoma cells. Whole midline sections of frozen 13 day old tumours were fixed and stained with anti-endomucin antibody. (**A**) The total number of blood vessels was counted across entire tumour sections and divided by the section area to give total mean blood vessel density for each genotype. (**B**) Graph showing the percentage of total blood vessels that are closed in tumour sections. (**C**) Graph showing mean numbers of lumenated vessels per mm^2^ of midline tumour section. (**D**) Representative images of endomucin-positive vessels in all genotypes *Arrows*, lumenated vessels; *arrowheads*, non-lumenated vessels. Scale bar 50 µm. N = 6 mice per genotype. For all graphs, bars show means ± SEM. NSD: no significant difference. * *P*<0.05.

Since tumour blood vessels appeared disrupted in Cldn14-het mice, but tumour size was not affected, we then examined the numbers of blood vessels in midline sections of size-matched tumours grown in WT, Cldn14-Het and Cldn-14 null mice. Surprisingly, blood vessel densities across midline tumour sections were found to be elevated in tumours grown in Cldn14-het mice when compared with WT and Cldn14-nulls (**Figure 4A**). In contrast, total blood vessel density was comparable between all genotypes in unchallenged skin suggesting that the change in tumour blood vessel density was induced specifically in the tumour environment (**Figure S2**). The apparent lack of correlation between tumour size and blood vessel density suggested that the functionality of the vessels in tumours grown in Cldn14-het mice might have been affected. Tumour blood vessel functionality is heterogeneous like the tumour mass itself, including those that are functional (lumenated), and those that are not (closed). Analysis of endomucin-stained tumour sections showed that the percentage of closed vessels was elevated in Cldn14-het tumour sections (**Figure 4B**), while lumenated vessel density was unchanged between tumours grown in WT, Cldn14-het or Cldn14-null mice (**Figure 4C**). Thus the lack of differences in lumenated blood vessels between genotypes correlates with the similar tumour growth rates in WT, Cldn14-het and Cldn14-null mice.

**Figure 5:**
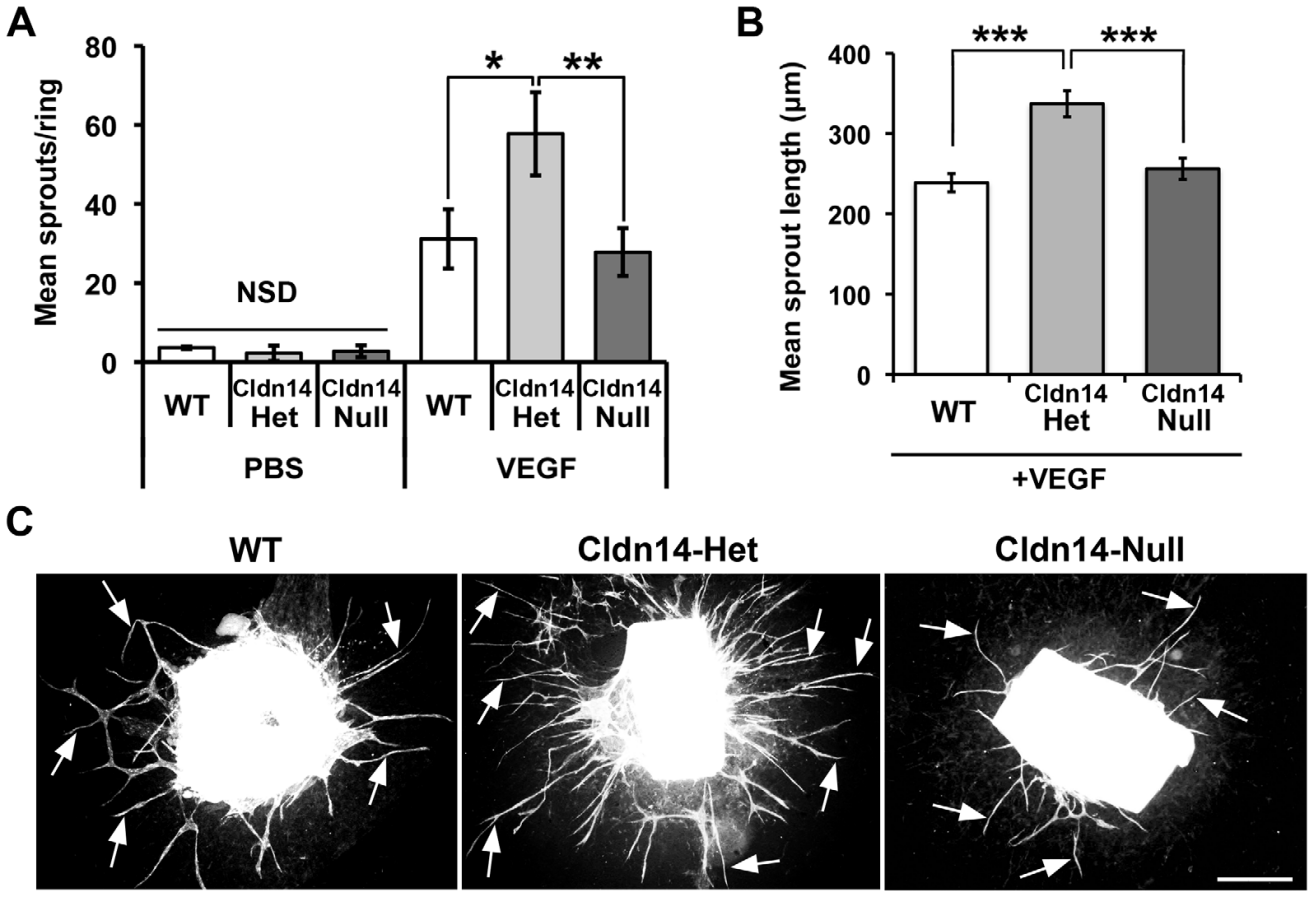
Cldn14 heterozygosity increases VEGF-stimulated aortic ring microvessel sprouting and sprout length. **A**. Quantitation of wild-type, Cldn14-heterozygous and Cldn14-null VEGF-stimulated aortic ring microvessel sprouting at 9 days in culture. PBS was used as a negative control. VEGF-stimulated microvessel numbers were increased significantly in Cldn14-het samples when compared with similarly treated WT and Cldn14 –null samples. **B**. Quantification of microvessel sprout length in using the ImageJ line tool on scaled images. N = 25–91 rings per genotype. VEGF-stimulated microvessel length was increased significantly in Cldn14-het samples when compared with similarly treated WT and Cldn14-null samples. **C**. Representative images of VEGF-treated BS1 lectin-stained aortic rings fixed and stained after 9 days in culture. *Arrows*, endothelial microvessel sprouts. Scale bar 500 µm. * *P*<0.05, ** *P*<0.01, *** *P*<0.001.

To examine further the effect of Cldn14-heterozygosity on angiogenic processes, the *ex vivo* aortic ring assay was used [24]. Aortic rings isolated from WT, Cldn14-het and Cldn14-null mice were embedded in collagen and treated with the pro-angiogenic factor, VEGF or PBS as a negative control. In the absence of VEGF microvessel outgrowth was minimal and the same across all genotypes (**Figure 5A**). However, VEGF stimulated an increase in microvessel outgrowth in both WT and Cldn14-null aortic rings and this was enhanced significantly in Cldn14-het aortic rings (**Figure 5A, C**). Moreover, microvessels were also significantly longer in Cldn14-het aortic ring assays when compared with WT or Cldn14-null tests as determined by BS1 lectin staining (**Figure 5B, C**). Taken together, the enhanced total tumour blood vessel density counts *in vivo* and increased microvessel sprouting *ex vivo* in Cldn14-hets indicate that partial, but not complete, loss of Cldn14 is sufficient to enhance angiogenic responses, but with compromised functionality.

### Claudin14 heterozygosity increases endothelial cell proliferation

**Figure 6:**
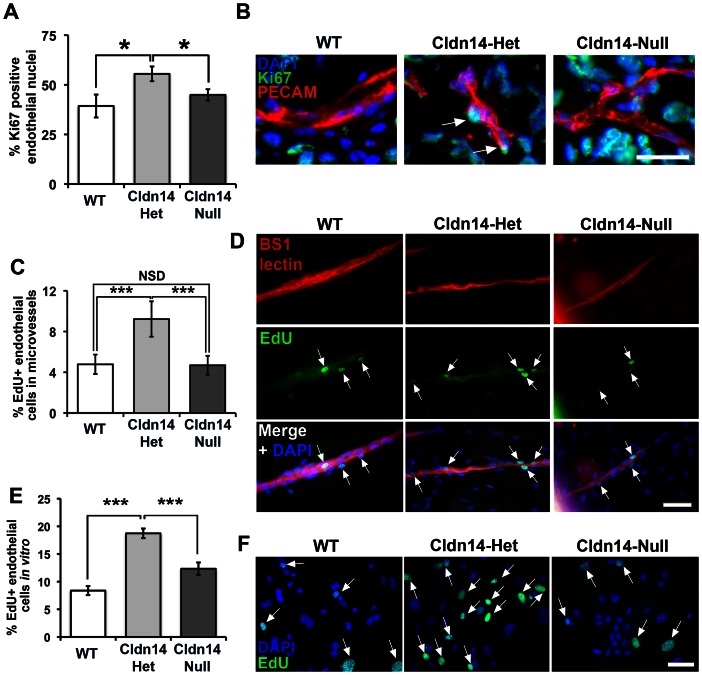
Cldn14 gene copy number affects endothelial cell proliferation *in vivo*, *ex vivo* and *in vitro*. (**A**) Percentages of Ki67-positive endothelial cells were counted in cryosections of 13-day B16F10 tumours from WT, Cldn14-het and Cldn14-null mice co-stained with PECAM. Endothelial cell proliferation was enhanced significantly in Cldn14-het mice. (**B**) Representative images of tumour sections in each genotype. *Arrows*, Ki67-positive endothelial cell nuclei. Scale bar 25 µm. (**C**) Proliferating cells in VEGF-stimulated wild-type, Cldn14-het and Cldn14-null collagen-embedded aortic explants were detected by EdU incorporation. The number of proliferating (EdU-positive) nuclei, counterstained with DAPI, was divided by the total number of cell nuclei also BS1-lectin positive to give % proliferating endothelial cells in VEGF-treated aortic rings. Bars show mean % of proliferating cells ± SEM. n = 6–8 rings per genotype, 513–717 nuclei per genotype. (**D**) Representative images of VEGF-stimulated WT, Cldn14-het and Cldn14-null microvessels from aortic ring explants stained for EdU and BS1 lectin. Scale bar 50 µm. (**E**) WT, Cldn14-het and Cldn14-null primary endothelial cells were examined for EdU incorporation in the presence of 30 ng/ml VEGF. Cells were counterstained with DAPI and the number of EdU-positive cells recorded for each genotype. Bars show mean % EdU-positive cells ± SEM. N = 1217–3464 nuclei per genotype, 3 mice per genotype. (**F**) Representative images of primary endothelial cells in culture. Scale bar 50 µm. *Arrows*, EdU-positive nuclei. NSD: no significant difference. * *P*<0.05, *** *P*<0.001.

Since enhanced angiogenic responses may reflect an increase in endothelial proliferation we hypothesized that Cldn14-hetrozygosity may elevate these processes. To test this, we first measured proliferation of endothelial cells in tumours *in vivo* (**Figure 6A, B**), *ex vivo* explants (**Figure 6C, D**) and cultured primary microvascular endothelial cells from the lungs (**Figure 6E, F**). Data revealed that proliferation was indeed enhanced *in vivo*, *ex vivo* and *in vitro* in Cldn14-het endothelial cells when compared to both WT and Cldn14-nulls (**Figure 6**). Enhanced angiogenesis may also reflect changes in endothelial migration. We therefore also analysed the ability of endothelial cells, isolated from each genotype, to migrate in a VEGF gradient [28]. Time-lapse and cell tracking analysis revealed a small decrease in the speed and persistence of cell movement of Cldn14-null cells compared to both WT and Cldn14-het cells (**Figure S3**).

These combined results demonstrate a role for the tight junction protein Cldn14 in maintenance of tumour blood vessel integrity and angiogenesis that was previously unknown. Importantly this effect is realised not by the total genetic ablation of Cldn14 but in its partial expression within the stromal microenvironment.

## Discussion

The consequences of changes in Cldn14 expression levels on tumour blood vessel fragility and angiogenesis have not been addressed previously. Here we have shown that Cldn14 heterozygosity, but not total deficiency, induces destabilisation of tumour blood vessels, which correlates with enhanced vessel leakage and decreased tumour hypoxia without affecting tumour growth.

Several papers have described how loss off cell-cell junction function can enhance blood vessel leakage [29]–[32]. For example, genetic ablation of VECAD and endothelial-specific deletion of the cytoplasmic associated signalling molecule β-catenin have reported a decrease vascular integrity, but most of these studies have been confined to phenotypes observed in null mutant mice [29], [30]. It may be that the lack of angiogenic phenotypes in the Cldn14-null mice is due to be due to molecular compensation, for example by other claudin family members expressed in tumour endothelial cells. Given that Cldn5 is an endothelial cell claudin [13], [15], [16], we tested for differences in Cldn5 mRNA levels in Cldn14-WT, Cldn14-het and Cldn14-null mouse kidney and brain samples by qPCR. We found no significant differences in the levels of Cldn5 message between the genotypes (data not shown) suggesting that Cldn5 compensation may not be the cause of the lack of Cldn14-null phenotypes. However, there may still be differences in protein levels in the tumour context that we were unable to identify in this study. The details of the mechanism by which this hypothesised compensation occurs is yet to be uncovered, but represents an important future goal for understanding potential co-regulation and crosstalk between levels of cell adhesion molecules during angiogenesis.

Our observations that Cldn14 heterozygosity, but not total deficiency, can cause: decreased endothelial cell-cell junctional organisation; poor blood vessel basement membrane distribution; and reduced supporting cell coverage, all describe how more subtle changes in endothelial cell-cell junctions can dramatically affect vascular function.

Claudins have been described to signal in co-ordination with β1-integrins. Genetic ablation of the α3-integrin subunit results in a basement membrane defect in which components of the basement membrane, such as laminin, show a disorganised expression pattern and a ‘shorelining effect’ [33] that is strikingly similar to that observed in the tumour blood vessels of Cldn14-het mice. In future studies, it would of interest to examine the effect of Cldn14-heterozygosity on α3β1-integrin expression and function since this may explain part of the phenotype observed here. This disruption of the basement membrane organisation may be the cause of the decreased supporting cell coverage in Cldn14-het tumour blood vessels. This notion is corroborated by previously published work in which mice lacking the laminin α4 chain displayed reduced pericyte recruitment to blood vessels [34]. Alternatively, the reduced pericyte coverage to Cldn14-het blood vessels could simply reflect a loss of cell-cell adhesion, either a knock-on effect of the decreased association between endothelial cells that subsequently affects pericyte adhesion, or even between endothelial cells and pericytes directly. In line with this idea, it has been reported that, in human glioblastoma multiforme patients, expression of claudins 1 and 5 is drastically reduced, together with an increase in blood vessel fragility and decreased α-SMA-positive differentiated pericyte coverage [35].

In addition, the increased tumour vascular fragility that we have observed in Cldn14-het mice is associated with enhanced endothelial proliferation *in vivo*, *ex vivo* and in endothelial cell cultures *in vitro*. It is tempting to speculate that this gives rise to an actual increase in total blood vessel numbers in Cldn14-het mice even if a significant proportion of these vessels are not properly lumenated. Cldn14 has previously been found to be downregulated in proliferating endothelial cells [36]. Our results may expand upon these findings, showing that partial loss of Cldn14 could be responsible for enhanced endothelial proliferation. It is known that tight junctions influence cellular proliferation by downstream signalling pathways including the transcription factor ZONAB, which can shuttle to the nucleus and interact with Cdk4. This pathway in turn regulates cyclin D1 and PCNA to influence G1 to S phase cell cycle progression [11], [37]. It may be that the partial loss of Cldn14 alters the available nuclear pool of ZONAB and affects cellular proliferation in this way; further studies of ZONAB subcellular localisation and proliferation markers in Cldn14-het endothelial cells could explore this possibility. How does this relate to the loss of lumen formation in a significant proportion of Cldn14-het tumour blood vessels? Reports have shown that lumen formation is caused by apoptosis [38]. It is therefore conceivable that an imbalance of proliferation and apoptosis in Cldn14-het endothelial cells is the reason for the reduced lumen formation in Cldn14-het mice. Our results indicate that since total loss of Cldn14 is unlikely to be physiologically relevant understanding the effects of partial loss of Cldn14 in the context of the endothelium, using a gene dosage strategy may be important in understanding the regulation of its biological functions.

Our work may also have some clinical relevance. It is difficult to prove that any pharmacological inhibitor has a complete blocking effect when used therapeutically, therefore understanding how partial loss, or blockade, may affect biological outcome also becomes of interest. To our knowledge Cldn14 inhibitors have not yet been tested for their effect on blood vessels, if indeed they are available, but if they were it is conceivable that partial pharmacological inhibition could promote some or all of the phenotypes that we have described in the Cldn14-het mice. For example, partial Cldn14 inhibition may decrease intratumoural hypoxia and pericyte association with blood vessels. Others have shown that decreased tumour hypoxia can sensitise tumour cells to chemotherapy and radiotherapy treatments [39]–[41] and that decreased pericyte coverage can increase sensitivity to anti-vascular agents [42], [43]. Thus, is it possible that combining anti-Cldn14 strategies with chemotherapy or anti-VEGF therapies may provide benefits [44]. All of these are certainly interesting ideas for future studies but beyond the scope of the report presented here.

In short, our findings highlight new roles for Cldn14 in vascular function and angiogenesis that are relevant directly to its levels of expression and not simply the presence of absence of this molecule.

## Supporting Information

Figure S1 **Genotyping and colony statistics for Cldn14 mice.** (**A**) A representative agarose gel is shown with separate PCR reactions, for Cldn14 WT (lanes 1, 3 and 5) and Cldn14-null alleles (lanes 2, 4 and 6). PCR products identify wild-type (lanes 1 and 2), Cldn14-heterozygous (lanes 3 and 4) and Cldn14-null (lanes 5 and 6) DNA samples. (**B**) The bar chart represent the numbers and of WT, Cldn14-Het and Cldn14-null mice at weaning from Cldn14 heterozygous breeding pairs. All genotypes developed at expected Mendelian ratios. N = 25 litters and 189 mice. (**C**) Proportion of male:female pups in the Cldn14 colony is normal and as expected. (**D**) qPCR analysis Cldn14 transcript expression from WT, Cldn14-het and Cldn14-null tissues. Cldn14 mRNA levels are shown relative to β-actin (ACTB) controls, with approximately half as much transcript detected in Cldn14-het organs and undetectable levels in Cldn14-nulls when compared with WT controls. Please see **Text S1** for qPCR method details. Bars show relative transcript levels ± SEM. N = 3 separate tissue samples per genotype.
(TIF)

Figure S2 **Vessel density in unchallenged skin is unaffected by the Cldn14 genotype.** Blood vessel density was quantified in WT, Cldn14-het and Cldn14-null transverse skin sections, taken from non-tumour burdened mice. Values are given as mean number of dermal blood vessels per mm^2^ of dermal section. Bars represent mean ± SEM. NSD: no significant difference.
(TIF)

Figure S3 **Migration of WT, Cldn14-het and Cldn14-null endothelial cells.** Primary endothelial cells were cultured from WT, Cldn14-heterozygous and Cldn14-null mouse lungs. Cells were plated on coverslips and inverted over Dunn chamber slides filled with serum-free growth medium and medium containing 100 ng/ml VEGF to stimulate cell movement. Cells were photographed at 10-minute intervals over 16 hours to create movie files for cell tracking with Andor software and analysis using Mathematica software. (**A**) Raw cell tracking data with all cell starting positions at a single point of origin. (**B**) Speed of cellular movement (µm/min). (**C**) Persistence of cell movement (tendency of cells to move directionally without deviation). Please see **Text S1** for Dunn Chamber Chemotaxis assay method details. Bar charts show means ± SEM. N = 12–20 fields per genotype, 280–348 cells per genotype, 2 independent experiments. * *P*<0.05 ** *P*<0.01.
(TIF)

Text S1 **Supplementary methods.** Details of methods used to produce data for **Figure S1** and **Figure S2**: organ harvesting, RNA extraction, qPCR and Dunn Chamber Chemotaxis assays.
(DOC)
