# Supplementary figures and images for: Stromal Claudin14-Heterozygosity, but Not Deletion, Increases Tumour Blood Leakage without Affecting Tumour Growth
Source: PLoS One. 2013 May 13;8(5):e62516. doi: 10.1371/journal.pone.0062516 (PMC3652830; doi:10.1371/journal.pone.0062516)

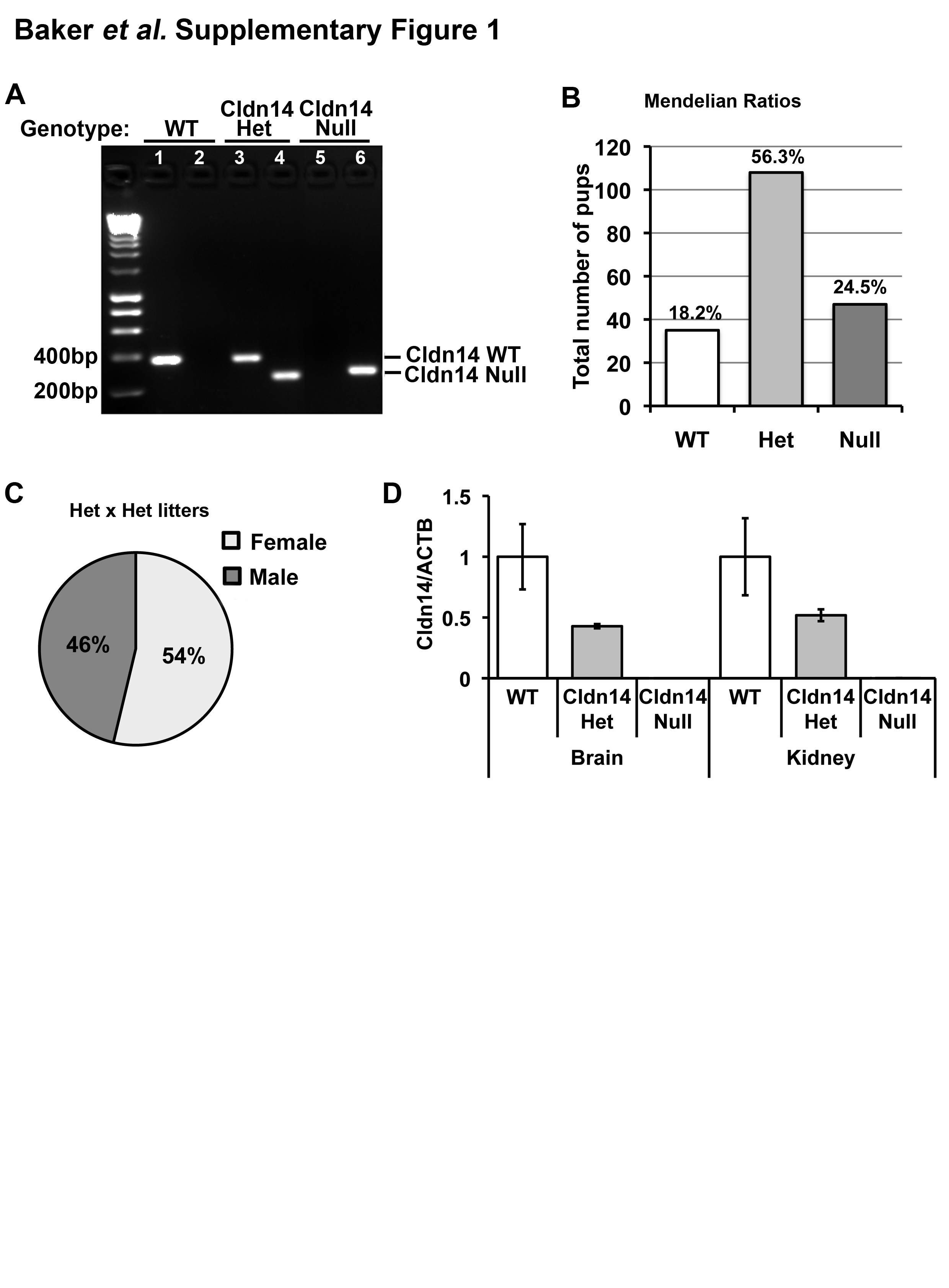

Supplement: Figure S1 — Genotyping and colony statistics for Cldn14 mice. (A) A representative agarose gel is shown with separate PCR reactions, for Cldn14 WT (lanes 1, 3 and 5) and Cldn14-null alleles (lanes 2, 4 and 6). PCR products identify wild-type (lanes 1 and 2), Cldn14-heterozygous (lanes 3 and 4) and Cldn14-null (lanes 5 and 6) DNA samples. (B) The bar chart represent the numbers and of WT, Cldn14-Het and Cldn14-null mice at weaning from Cldn14 heterozygous breeding pairs. All genotypes developed at expected Mendelian ratios. N = 25 litters and 189 mice. (C) Proportion of male:female pups in the Cldn14 colony is normal and as expected. (D) qPCR analysis Cldn14 transcript expression from WT, Cldn14-het and Cldn14-null tissues. Cldn14 mRNA levels are shown relative to β-actin (ACTB) controls, with approximately half as much transcript detected in Cldn14-het organs and undetectable levels in Cldn14-nulls when compared with WT controls. Please see Text S1 for qPCR method details. Bars show relative transcript levels ± SEM. N = 3 separate tissue samples per genotype. (TIF) [file pone.0062516.s001.tif]

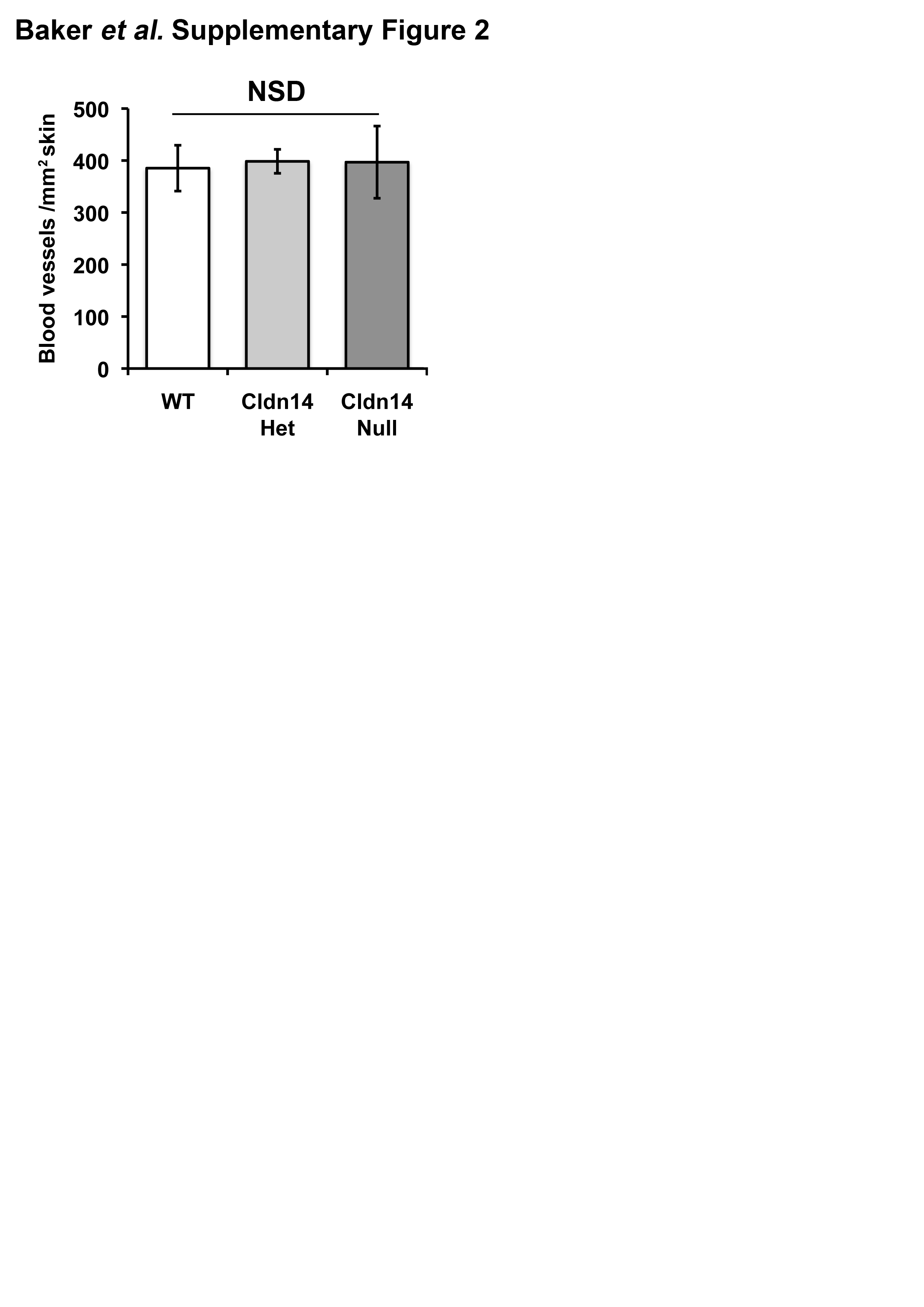

Supplement: Figure S2 — Vessel density in unchallenged skin is unaffected by the Cldn14 genotype. Blood vessel density was quantified in WT, Cldn14-het and Cldn14-null transverse skin sections, taken from non-tumour burdened mice. Values are given as mean number of dermal blood vessels per mm2 of dermal section. Bars represent mean ± SEM. NSD: no significant difference. (TIF) [file pone.0062516.s002.tif]

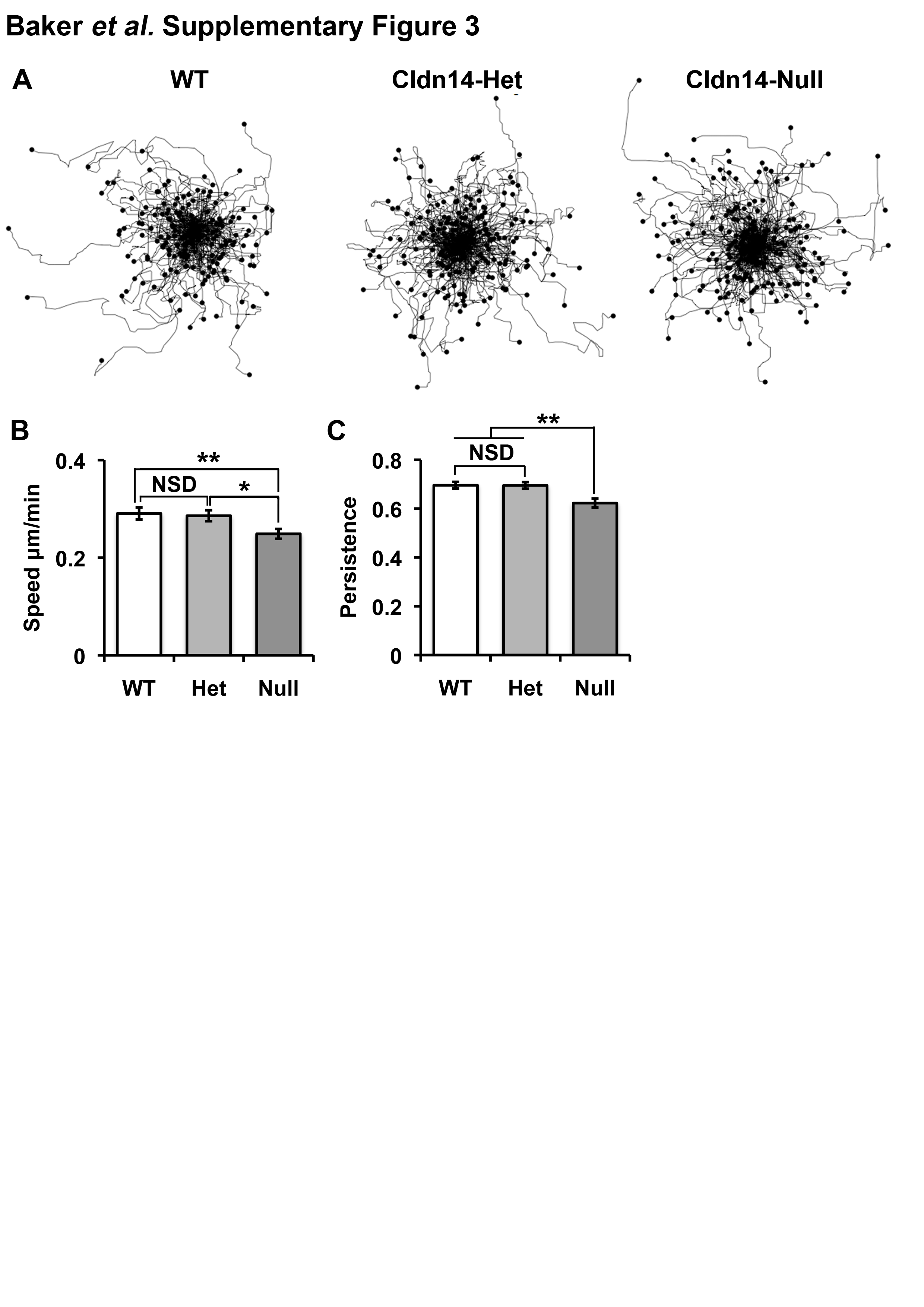

Supplement: Figure S3 — Migration of WT, Cldn14-het and Cldn14-null endothelial cells. Primary endothelial cells were cultured from WT, Cldn14-heterozygous and Cldn14-null mouse lungs. Cells were plated on coverslips and inverted over Dunn chamber slides filled with serum-free growth medium and medium containing 100 ng/ml VEGF to stimulate cell movement. Cells were photographed at 10-minute intervals over 16 hours to create movie files for cell tracking with Andor software and analysis using Mathematica software. (A) Raw cell tracking data with all cell starting positions at a single point of origin. (B) Speed of cellular movement (µm/min). (C) Persistence of cell movement (tendency of cells to move directionally without deviation). Please see Text S1 for Dunn Chamber Chemotaxis assay method details. Bar charts show means ± SEM. N = 12–20 fields per genotype, 280–348 cells per genotype, 2 independent experiments. * P<0.05 ** P<0.01. (TIF) [file pone.0062516.s003.tif]
