# Supplementary material for: Stromal Claudin14-Heterozygosity, but Not Deletion, Increases Tumour Blood Leakage without Affecting Tumour Growth
Source: PLoS One. 2013 May 13;8(5):e62516. doi: 10.1371/journal.pone.0062516 (PMC3652830; doi:10.1371/journal.pone.0062516)
Supplement: Text S1 — Supplementary methods. Details of methods used to produce data for Figure S1 and Figure S2: organ harvesting, RNA extraction, qPCR and Dunn Chamber Chemotaxis assays. (DOC) [file pone.0062516.s004.doc]

### SUPPLEMENTARY METHODS

**Quantitative PCR**

Organs were harvested from WT, Cldn14-het and Cldn14-null mice and snap-frozen in liquid N_2_ then stored at -80 °C. Samples were weighed and ground to powder in liquid N_2_. Samples for RNA extraction were homogenised and spun for 3 minutes at 13,000 g and the supernatant collected, frozen on dry ice and stored at -80 °C. The Qiagen RNeasy mini kit, with a DNaseI digestion step, was used to extract RNA from the tissue supernatants, according to the manufacturer’s instructions. Samples were eluted in RNase-free H_2_O and passed twice through the final elution columns. RNA concentration was analysed using a ND-1000 Spectrophotometer (Nanodrop, Delaware, USA). For cDNA generation, the High-Capacity cDNA Reverse Transcription Kit (Applied Biosystems) was used according to the manufacturer’s instructions, but without RNAse inhibitor. 1.5 μg RNA was used per 20 μl reaction volume. Pre-designed primers for Cldn14 were purchased from Origene (qSTAR expression detection system). β-actin primers were used as controls: ACTB forward: 5’ – AAG GCC AAC CGT GAA AAG AT – 3’. ACTB reverse: 5’ – GTG GTA CGA CCA GAG GCA TAC – 3’. Reactions were performed with the SYBR Green PCR master mix (Applied Biosystems) in optical qPCR plates (Applied Biosystems). To amplify Cldn cDNA: 0.8 μl primers were used, as recommended by Origene, with 100 ng cDNA. 1 μl (50 nM) ACTB primers were used with 10 ng cDNA.

**Dunn Chamber chemotaxis assays**

Primary endothelial cells grown in culture as described previously were seeded on square glass coverslips (Hawksley 22 mm x 25 mm x 0.17 mm cover glass, Z50000) and serum-starved in OptiMEM® overnight. Coverslips were then inverted over glass slides containing consecutive circular chambers (Hawksley Chemotaxis Dunn counting chamber, DC100) filled with OptiMEM®, and the outer chamber supplemented with 100 ng/ml VEGF. Coverslips were sealed using a wax mixture (1:1:1 mixture of beeswax, paraffin wax and petroleum jelly). Fields were selected for overnight image capture at intervals of 10 minutes over 16 hours. Movie files were then imported into Andor IQ acquisition software (Andor Bioimaging, Belfast, Northern Ireland) to obtain individual cell track data from each movie. Resulting cel files were then analysed in Mathematica™ software (v6, Wolfram Research, Oxfordshire, UK) using the Dunn chamber Notebook (written by Prof. Graham Dunn, Kings College London). Instances of apparent cell division and cell death were also quantified visually from the movie files.
